# Genome-wide association study (GWAS) identifies genetic loci controlling Distinctness, Uniformity, and Stability (DUS) traits in wheat

**DOI:** 10.1007/s00122-025-05130-4

**Published:** 2026-01-28

**Authors:** Camila M. Zanella, Richard Horsnell, Bethany Love, Tally I. C. Wright, Jutta Taferner-Kriegl, Clemens Flamm, Lorella Andreani, Chiara Delogu, Vanessa McMillan, Margaret Wallace, Elizabeth Scott, James Cockram

**Affiliations:** 1 Niab, Park Farm, Villa Road, Histon, Cambridge, CB24 9NZ UK; 2 Austrian Agency for Health and Food Safety (AGES), Spargelfeldstraße 191, 1220 Vienna, Austria; 3 Council for Agricultural Research and Economics - Research Centre for Plant Protection and Certification, Tavazzano, LO Italy

## Abstract

**Key message:**

Genetic analysis of wheat Distinctness, Uniformity, and Stability (DUS) characteristics identifies significant marker–trait associations for 15 DUS traits, including associations for ‘seed: colouration with phenol’ closely linked to *Ppo-A1*.

Crop improvement via breeding underpins the yield gains required for future food security. Commercial development of new varieties is supported by the legal protection afforded by Plant Variety Rights, which for wheat is awarded via evaluation of 27 morphological ‘characteristics’ as part of Distinctness, Uniformity, and Stability (DUS) testing. While identification of molecular markers predictive for wheat DUS characteristics would be useful to aid processes such as variety identification and DUS test optimization, little is known about their genetic control. Here we assemble a panel of 412 European wheat varieties, along with corresponding DUS phenotypic data and genotype using the TaNG 43k genotyping array. The resulting 14,921 polymorphic genetic markers were distributed approximately evenly between the A (33%), B (38%), and D (29%) wheat sub-genomes. DUS characteristic heritability (*h*^2^) varied (mean = 0.44), ranging from 0.27 (‘ear: glaucosity’) to 1.00 (‘ear: scurs or awns’). Subsequent genome-wide association study (GWAS) identified significant marker–trait associations for 15 of the 24 DUS characteristics analysed, resolved into 57 genetic loci. Of note, a highly significant association (− log_10_P = 44.79) for ‘seed: colouration with phenol’ on chromosome 2A was just 0.37 Mb from the *Ppo-A1* gene known to control discolouration in wheat food products. GWAS was less successful for DUS characteristics with low heritability (traits with *h*^*2*^ below or above 0.5 had a mean of 1.2 and 5.3 GWAS hits, respectively), for which the use of molecular markers would be more suited to alternative approaches such as genomic prediction. Collectively, this work will inform marker-aided approaches for DUS-relevant applications.

**Supplementary Information:**

The online version contains supplementary material available at 10.1007/s00122-025-05130-4.

## Introduction

Global food security projections indicate food demand will increase by at least 30% between 2010 and 2050 (van Dijk et al. 2021). Improvements to crop production via the use of better performing varieties, as well as improvements to agronomy, are essential to help meet future food security. For example, studies of European cereal and oil seed rape production show that over 88% of yield improvement between 1982 and 2011 is due to genetic improvement (Mackay et al. 2011). Such analyses highlight the critical role of plant breeding in delivering food security. Developing new plant varieties is typically a costly and time-consuming process. To help ensure return on such investments, a widely used framework for breeder protection of their new varieties is Plant Breeders’ Rights (PBR), issued under the International Union for the Protection of New Varieties of Plants (UPOV) Convention 1991. In accordance with this Act, protected varieties are granted PBR for a period of no less than 20 years (UPOV 1991). The PBR system is based on a predominantly morphological description of a plant variety, using a set of phenotypic traits defined and regulated by UPOV (Jones et al. 2013). For the rights of a breeder to be protected in any of the 80 UPOV signatory member states or regions, their plant variety must be shown to be morphologically Distinct from all other common knowledge varieties, and the expression of these traits must be sufficiently Uniform and Stable as determined by assessment across at least two growing cycles. The central position of DUS for the subsequent release and sale of new improved agricultural crop varieties means that Distinctness, Uniformity, and Stability (DUS) traits and their assessment form a key part of the pipeline that flows from applied research to crop breeding, varietal release, and on-farm production. Therefore, understanding of the genetic control of DUS characteristics has implications and potential uses in a variety of settings. These include assessing varietal purity, aiding variety identification, and optimization of the DUS testing process itself (Jamali et al. 2019). Indeed, the use of molecular markers within the DUS system was the focus of the UPOV advisory group on ‘Biochemical and Molecular Techniques and DNA profiling in Particular’ (BMT) and is now included in the work of the Technical Working Group on testing methods and techniques (TWM).

Within the temperate cereal crops, systematic analysis of DUS characteristics has been undertaken previously in the diploid grass species barley (*Hordeum vulgare* L.), allowing subsequent generation of molecular markers for use in downstream applications (e.g. Cockram et al. 2010, 2012; Yang et al. 2021). This included analysis of molecular markers for their ability to determine ‘off-types’ within DNA extracted from multiple individuals (Saccomanno et al. 2020), required to address Uniformity within DUS processes. Genetic analysis of DUS characteristics in wheat (*Triticum aestivum* L., the world’s third most important cereal crop) is much less well studied, due possibly to the size and complexity of its 17-Gb hexaploid genome (2*n* = 6*x* = 42, AABBDD sub-genomes). UPOV currently describes 27 ‘DUS characteristics’ for wheat (UPOV guideline TG/3/12 Rev.), listed in Supplementary Table S1. These are measured at specific growth stages (GS) (Zadoks et al. 1974), ranging from dry seed (GS 0; ‘seed: colour’ and ‘seed: colouration with phenol’) to GS 80-92 (after the late milk stage; 13 DUS characteristics relating to the ear, glume, rachis, and straw morphology). While we are not aware of previous studies that have systematically analysed wheat DUS traits, some genetic loci of relevance to DUS have been map-based cloned. For example, it could be expected a priori that the DUS characteristic ‘ear: scurs or awns’ is largely determined by allelic variation at the genetic locus *Tipped 1* (*B1*) that determines the presence or absence of wheat awns, and which has been found to encode a C2H2 zinc finger transcription factor (Huang et al. 2020). Similarly, the DUS characteristic ‘seasonal type’ that differentiates varieties that require vernalization (winter types), those that don’t (spring types), and those that don’t require vernalization but are cold hardy (alternative types), could be expected to be largely determined by allelic variation at one or more of the vernalization response loci *VRN-A1, -B1* and *-D1*, encoded by homoeologous MADS-box transcription factors (e.g. Alonso-Peral et al. 2011; Díaz et al. 2012; Fu et al. 2005. Recently reviewed by Milec et al. 2023). Lastly, while all modern cultivars are expected to be semi-dwarf in stature, the DUS characteristic ‘plant: length’ could be influenced by known semi-dwarfing alleles at the *Rht-B1* and *-D1* loci which are encoded by homoeologous DELLA proteins (Peng et al. 1999).

To address current knowledge gaps in the genetic architecture of wheat DUS characteristics, here we use genome-wide association study (GWAS) to systematically investigate the genetic control of DUS traits in wheat. Our aims were to (1) collate an association mapping panel of > 400 European wheat cultivars with associated DUS characteristic data, (2) genotype the panel with a high-density single-nucleotide polymorphism (SNP) array, and (3) identify significant marker–trait association for wheat DUS characteristics.

## Methods

### Wheat germplasm and DUS characteristic phenotypic data

Plant material (with variety name anonymized) and corresponding Distinctness, Uniformity, and Stability (DUS) phenotypic data for a panel of 458 wheat varieties were received from the five organizations that carry out DUS testing in the UK (Niab), Germany (Bundessortenamt), Hungary (National Food Chain Safety Office), Italy (Council for Agricultural Research and Economics), and Austria (Austrian Agency for Health and Food Safety). For the DUS characteristics investigated (Table 1), phenotypic data were collected during the Official DUS test for the variety (historical data) and were scored according to the Protocol for DUS tests for *Triticum aestivum* L. (Council Regulation 2100/94 on Community Plant Variety Rights). The scoring system for each characteristic is listed in Supplementary Table S1. DUS characteristics with more than 20% missing data or that showed very little meaningful variation in character states were removed from forward analyses. Estimates of heritability (*h*^2^) were determined using the standard univariate mixed linear model to partition the phenotypic variance for each DUS trait into additive genetic and residual variances. The model details are similar to a previously used model for estimating genomic heritabilities in barley DUS traits (Yang et al. 2021). Model fitting was undertaken using the *mmer* function in the package `sommer' (Covarrubias-Pazaran 2016) in RStudio (RStudio Team 2020). The *vpredict* function in the same package was used to obtain the estimates and standard errors for *h*^*2*^, which were calculated as a ratio of additive genetic variance over phenotypic variance (sum of additive genetic and residual variances). Histograms of DUS characteristic states were made using the package `ggplot2' (Wickham 2016) in RStudio.

**Table 1:** Wheat DUS characteristics

Trait code	UPOV code	DUS characteristic	No. cvs	*h*^2^	Included in GWAS
Trait 1	1	Seed: colour	408	0.64	Yes
Trait 2	2	Seed: colouration with phenol	408	0.56	Yes
Trait 3	3	Coleoptile: anthocyanin colouration	397	0.38	Yes
Trait 4	4	Plant: growth habit	411	0.33	Yes
Trait 5	5	Plant: frequency of plants with recurved flag leaves	408	0.51	Yes
Trait 6	6	Flag leaf: anthocyanin colouration of auricles	8	NA	No^$^
Trait 7	7	Time of ear emergence	412	0.30	Yes
Trait 8	8	Flag leaf: glaucosity of sheath	409	0.33	Yes
Trait 9	9	Flag leaf: glaucosity of blade	394	0.35	Yes
Trait 10	10	Ear: glaucosity	412	0.27	Yes
Trait 11	11	Culm: glaucosity of neck	412	0.37	Yes
Trait 12	12	Lower glume: hairiness on external surface	213	NA	No^&^
Trait 13	13	Plant: length	412	0.33	Yes
Trait 14	14	Straw: pith in cross sections	411	0.35	Yes
Trait 15	15	Ear: density	412	0.40	Yes
Trait 16	16	Ear: length	412	0.48	Yes
Trait 17	17	Ear: scurs or awns	409	1.00	Yes
Trait 18	18	Ear: length of scurs or awns	409	0.69	Yes
Trait 19	19	Ear: colour	410	NA	No^&^
Trait 20	20	Ear: shape in profile	412	0.34	Yes
Trait 21	21	Apical rachis segment: area of hairiness on convex surface	401	0.29	Yes
Trait 22	22	Lower glume: shoulder width	412	0.28	Yes
Trait 23	23	Lower glume: shoulder shape	412	0.31	Yes
Trait 24	24	Lower glume: length of beak	412	0.69	Yes
Trait 25	25	Lower glume: shape of beak	412	0.30	Yes
Trait 26	26	Lower glume: area of hairiness on internal surface	412	0.42	Yes
Trait 27	27	Seasonal type	409	0.70	Yes

The number of cultivars in the association mapping panel with DUS data is indicated, along with heritability (*h*^*2*^). Three DUS characteristics not included in genetic analyses, due either to: ^$^lack of cultivars with DUS data, or ^&^low trait variation

*NA* not applicable

### DNA extraction and genotyping

One seed per plant was sown into 96-well potting trays filled with compost and grown to the three-leaf stage in a temperature-controlled glasshouse. Leaf tissue was harvested for DNA extraction using the Fulton et al. (1995) method and DNA quality measured using a NanoDrop Spectrophotometer (ThermoFisher Scientific) following the manufacturer’s instructions. Genotyping was undertaken under subcontract at the Bristol Genomics Facility (University of Bristol, UK) using the Axiom *Triticum aestivum* Next Generation (TaNG) single-nucleotide polymorphism (SNP) array v1.1 (Burridge et al. 2024) with genotype calls made using the Axiom Analysis Suite software (ThermoFisher Scientific). All genotypes were scored as 0 (AA) or 1 (BB), with heterozygotes (AB) treated as missing data. The resulting genotypic dataset was processed to remove markers with missing data ≥ 10% and markers with a minor allele frequency ≤ 3%; the remaining missing values in the genotypic data were imputed in RStudio using the package `*miss*Forest' (Stekhoven and Büehlmann 2012) with 200 trees, which fits a random forest (Breiman 2001) on the observed genotypic data to predict the missing SNP data. The physical map locations of array SNPs on the wheat reference genome assembly of cv. Chinese Spring were obtained from Burridge et al. (2024). The markers were ‘skimmed’ to remove one of each pair of SNPs that were 100% correlated with each other (an absolute correlation coefficient r = 1), using a custom R script.

### Characteristics of the association mapping panel

Linkage disequilibrium (LD) decay along each chromosome and across the entire genome was investigated via pairwise correlation (*r*^2^) between all markers to find the pair that yielded the highest *r*^2^. LD analysis was performed in R using the `sommer' package. LD decay was determined by plotting the *r*^2^ values against physical distance (Mbp) in the wheat reference genome of cv. Chinese Spring RefSeq v1.0 (IWGSC 2018), and for each of the A, B and D sub-genomes a trend line was calculated by locally weighted polynomial regression (LOESS) curve in R using a decay threshold of *r*^2^ = 0.2. Principal component analysis (PCA) was conducted in RStudio using the package `stats' v.4.4.1 (R Core Team 2024) with 4252 markers that had been ‘skimmed’ to remove a SNP in each pair with an absolute correlation of *r* ≥ 0.6.

### Genetic analyses

The genotypic and phenotypic datasets were cross referenced; a total of 412 wheat cultivars were used for GWAS analysis and 24 DUS characters. GWAS was performed in RStudio v4.4.1 using the package `GWASpoly' (Rosyara et al. 2016) which implements a Mixed Linear Model (MLM). Population genetic stratification was accounted for using (i) population structure as a fixed effect, via the use of 5 principal components (PCs), and (ii) kinship as a random effect, determined using a subset of 4252 SNPs skimmed from the final genotypic dataset using a correlation threshold of r^*2*^ = 0.60. To identify significant MTAs, two thresholds were used: the false discovery rate (FDR) (Benjamini & Hochberg 1995) with a *q*-value cut-off of *q* = 0.05 and the more stringent Bonferroni corrected *p* = 0.05 threshold. MTAs were considered genuine when they had a significant score above the calculated threshold. For each trait in turn, significant SNPs on the same chromosome were treated as representing the same genetic locus if (a) they were a within 40 Mbp of the next significant SNP for the same trait, or (b) if they were located within the pericentromeric regions spanning the wheat centromeres, as identified in European wheat cultivars by Gardner et al. (2016). Groups of two or more quantitative trait loci (QTL) were subsequently deemed to be in a QTL cluster following the same approach. GWAS results were drawn in a chromosomal ideogram using R package `LinkageMapView' (Ouellette et al. 2018). For selected SNPs from the 43k array, conversion to the Kompetitive Allele-Specific PCR (KASP) molecular marker genotyping platform was undertaken as previously described (Gardner et al. 2025). The resulting KASP primers (Supplementary Table S2) were used to genotype an independent validation panel of 122 wheat cultivars, and boxplots of the resulting data were plotted using `ggplot2' with significance of allele class differences determined using the Mann–Whitney test in RStudio. Chromosome 2A haploblock and haplotype analysis using the genetic markers from the SNP array was conducted as described by Gardner et al. (2025).

## Results

### Features of the wheat association mapping panel

**Fig. 1:**
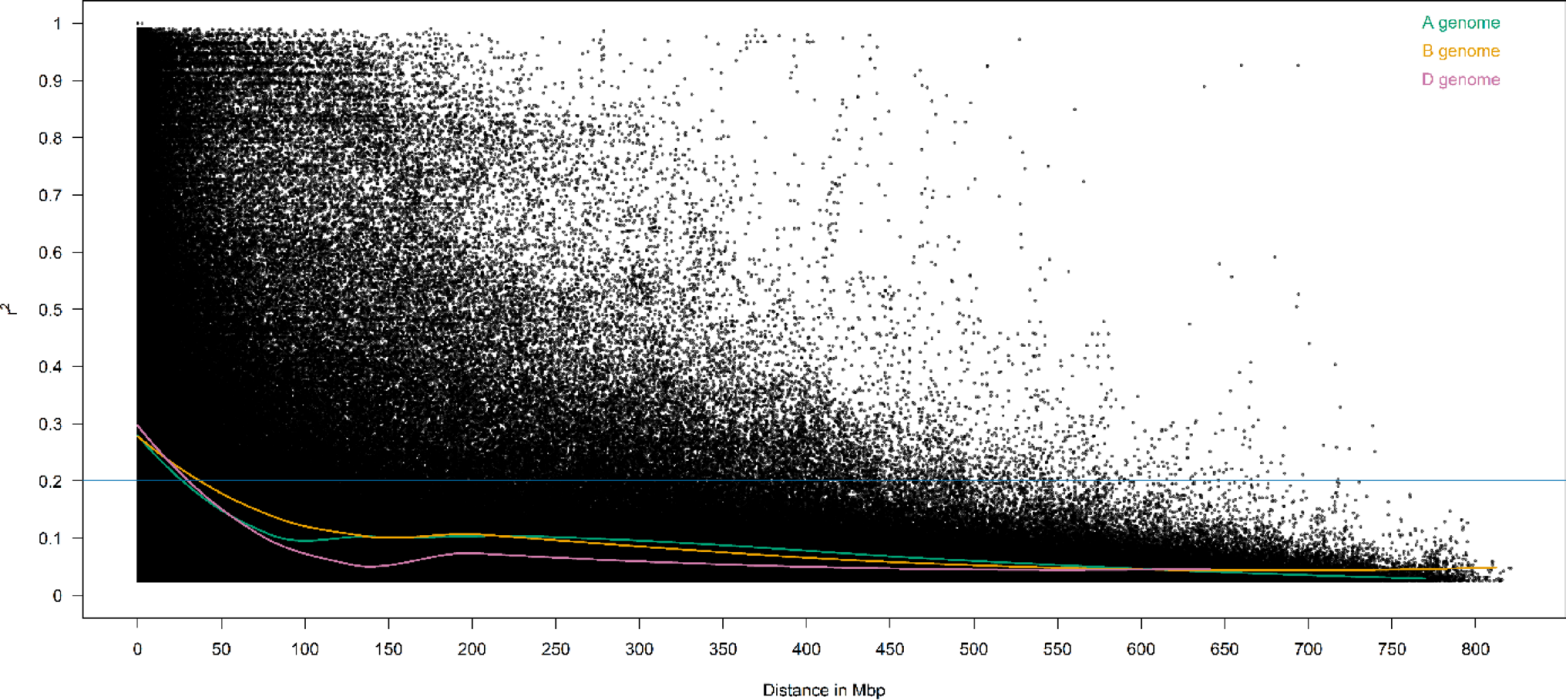
Plot of linkage disequilibrium (LD) decay in the association mapping panel (number of cultivars = 412, number of genetic markers = 4,252). A locally weighted polynomial regression (LOESS) curve has been fitted for markers on the A, B and D sub-genomes of wheat. The intersection between each LOESS curve and *r*.^*2*^ = 0.2 was used to report LD decay rates for each sub-genome: A (28 Mb), B (38.5 Mb), D (31.6 Mb)

**Fig. 2:**
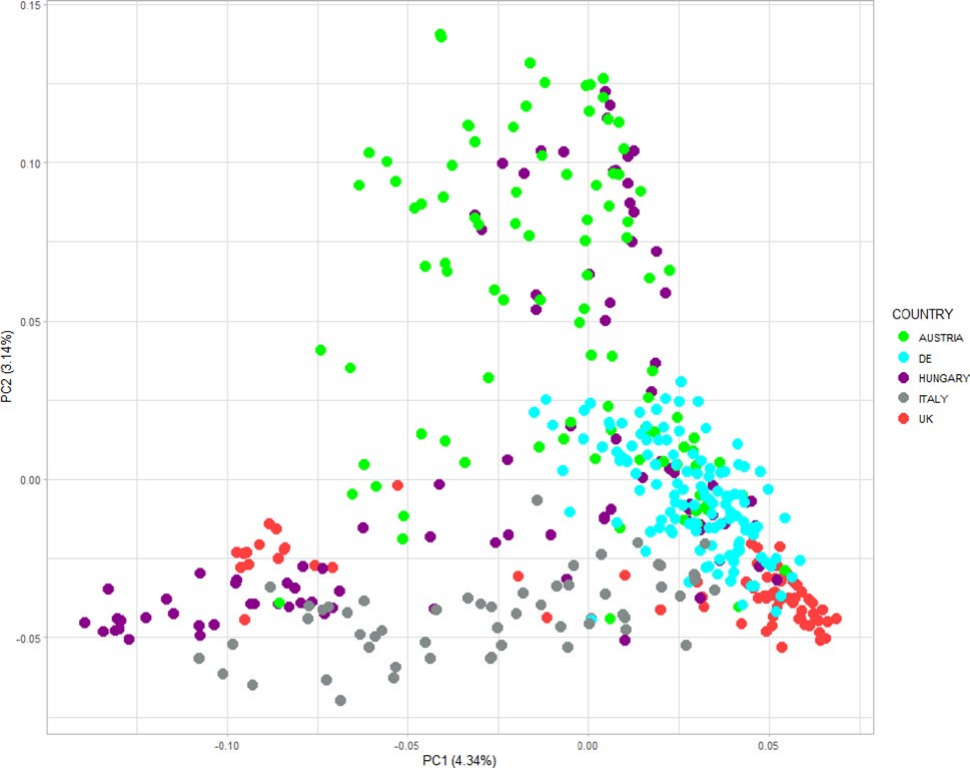
Investigation of genetic stratification in the ‘INVITE’ wheat association mapping panel (*n* = 412) via principal component analysis (PCA). Each datapoint shown represents a cultivar plotted in PCA space against principal components 1 (PC1) and PC2. The total percentage of the PCA variation each PC accounts for is indicated in brackets on the axis labels. Cultivar country of origin is as shown in the key

A wheat association mapping panel consisting of 458 cultivars was sourced from DUS Examination Offices in five countries: Austria, Germany, Hungary, Italy, and the UK (Table 2). Genotyping the panel using the Axiom *Triticum aestivum* Next Generation (TaNG) 43k array resulted in 43,373 SNPs successfully genotyped across 446 cultivars. The dataset was then quality controlled to remove markers with missing data ≥ 10% or minor allele frequency ≤ 3%, and cultivars with > 10% missing data or > 98% similarity based on genetic marker profile, resulting in 19,602 SNPs genotyped across 412 cultivars. Lastly, SNPs with 100% correlation were filtered so that only one from each correlated set was retained, resulting in a final genotypic dataset of 412 cultivars genotyped with 14,921 SNPs (Supplementary Table S3). The mean number of SNPs per chromosome was 711. The proportions of SNPs across the three wheat sub-genomes were marginally higher on the A (33%) and B (38%) sub-genomes than on the D (29%) (Supplementary Table S3). The extent of linkage disequilibrium (LD) between markers within chromosomes was determined by plotting LD against physical map distance and fitting a LOESS curve for each of the three wheat sub-genomes, finding LD decay rate to be similar on the A, B and D sub-genomes (28.0, 38.5, and 31.6 Mb, respectively) (Fig. 1). Next, principal component analysis (PCA) was undertaken to transform potentially correlated variables into a smaller set of variables (principal components, PCs) to investigate potential genetic stratification in the association mapping panel (Fig. 2). Plotting PC1 versus PC2 found stratification to be present, accounting for around 4% and 3% of the variation, respectively. Overlaying country of origin information found German cultivars to be the most prominently clustered in PC space. Cultivars from the four remaining countries were predominantly grouped due to PC1, with the exception of those from Austria and Hungary which were notably differentiated according to PC2.

**Table 2:** Summary information for germplasm in the ‘INVITE’ wheat association mapping panel

Country	Testing Centre/source	No. cultivars	No. cultivars with DUS data	No. cultivars with genotypic and DUS data
Austria	AGES	102	88	80
Germany	BSA	129	128	119
Hungary	NEBIH	91	91	86
Italy	CREA	56	56	54
UK	NIAB	80	79	73
**Total**		**458**	**442**	**412**

### Wheat DUS characteristics

**Fig. 3:**
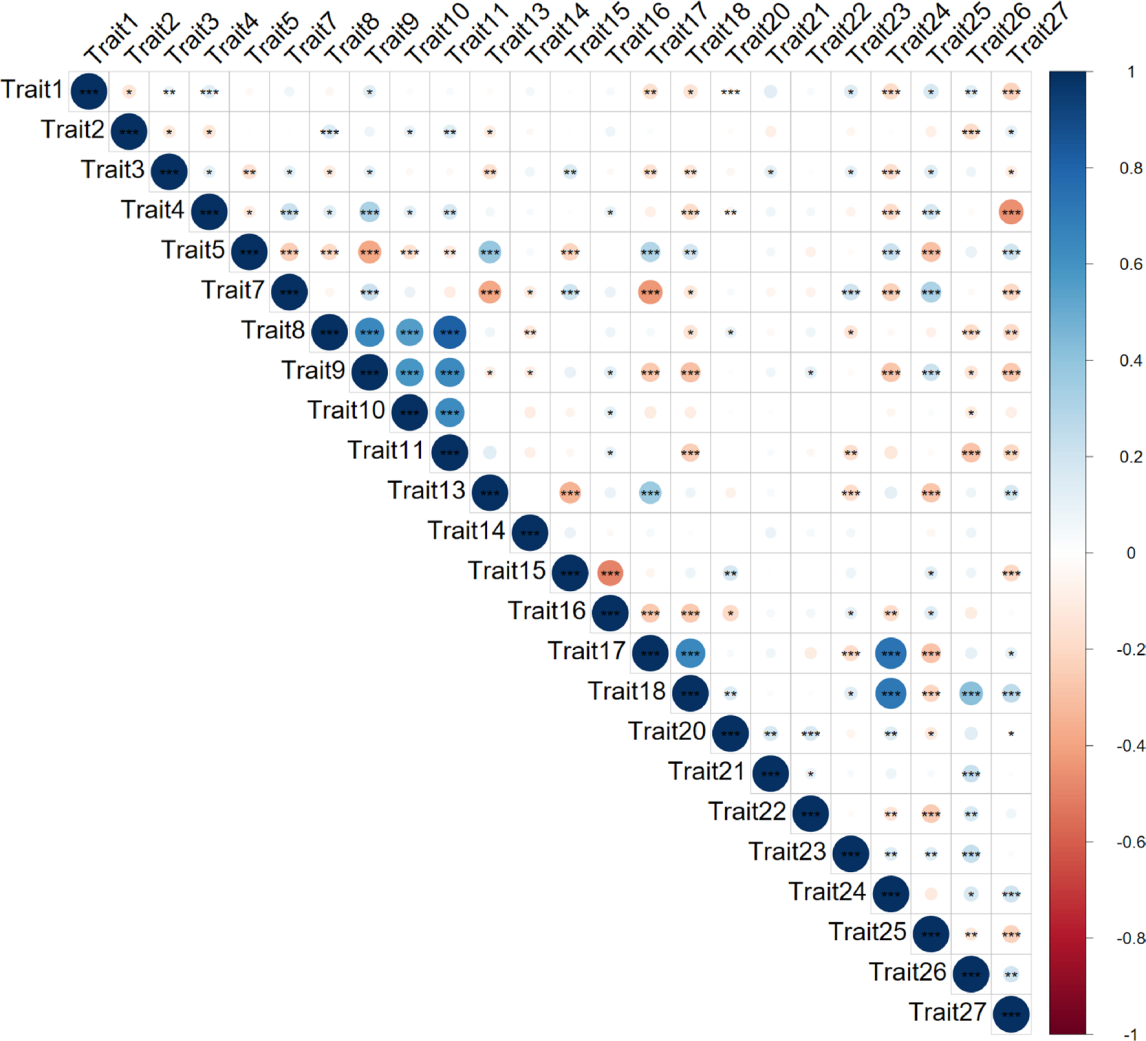
Correlations (r^2^) between DUS characteristics. Significant correlations at ^*^ *P* = 0.05, ^**^ *P* = 0.01, and ^***^ *P* = 0.001 determined by paired Pearson correlation coefficient are indicated

For the panel of 412 genotyped cultivars, phenotypic data for up to 27 DUS characteristics were sourced from official DUS records (Table 1; Supplementary Fig. S1). Of these, three characteristics were not used for subsequent analyses due either to low numbers of cultivars with data (Trait 6) or extremely low phenotypic variation (Traits 12 and 19). Of the remaining 24 wheat DUS characteristics, those scored using a more quantitative scale (with five or more ‘notes’ on the phenotypic scale) showed relatively normal trait score distributions—with the exception of Trait 18 (‘ear: length of scurs or awns’) which was bimodal (Supplementary Fig. S1). Of the three DUS characteristics scored using just three phenotypic ‘notes’: for Trait 14 (‘straw: pith in cross section’), 86% per cent of cultivars were scored as ‘thin’, with the remaining 14% of cultivars split relatively evenly between ‘medium’ and ‘thick’. For Trait 17 (‘ear: scurs or awns’), just 2% of cultivars were recorded as ‘absent’, while the sequentially increasing length of the structures extending from the glume tips represented by ‘scurs present’ and ‘awns present’ were present in 63% and 35% of cultivars, respectively. Finally, for Trait 27 (‘seasonal type’), 91% of cultivars were ‘winter’ type, while the progressively earlier flowering in the absence of vernalization scores of ‘alternative’ and ‘spring’ were present in 1% and 7% of the cultivars, respectively. Correlations were found between DUS characteristics, with both the mean and median number of significant (*P* < *0.05*) correlations per trait = 12 (Fig. 3). Notable negative correlations included that between ‘seasonal type’ (Trait 27) and ‘plant: growth habit’ (Trait 4), indicating that winter cultivars were more prostrate than spring types, and between ‘ear density’ (Trait 15) and ‘ear: length’ (Trait 16). The presence of awns (Trait 17) and the length of the lower glume beak (Trait 24) were found to be strongly positively correlated. Similarly, strong positive correlations were observed between the four DUS characteristics related to glaucosity in the flag leaf sheath (Trait 8) and blade (Trait 9), the ear (Trait 10) and culm (Trait 11) (*p* < 0.001). Overall, DUS characteristics were found to be moderately heritable (*h*^*2*^) (mean = 0.44, median = 0.36), ranging from 0.27 (Trait 10, ‘ear: glaucosity’) up to 1.00 (Trait 17, ‘ear: scurs or awns’) (Table 1, Supplementary Table S1).

### GWAS of wheat DUS characteristics

**Fig. 4:**
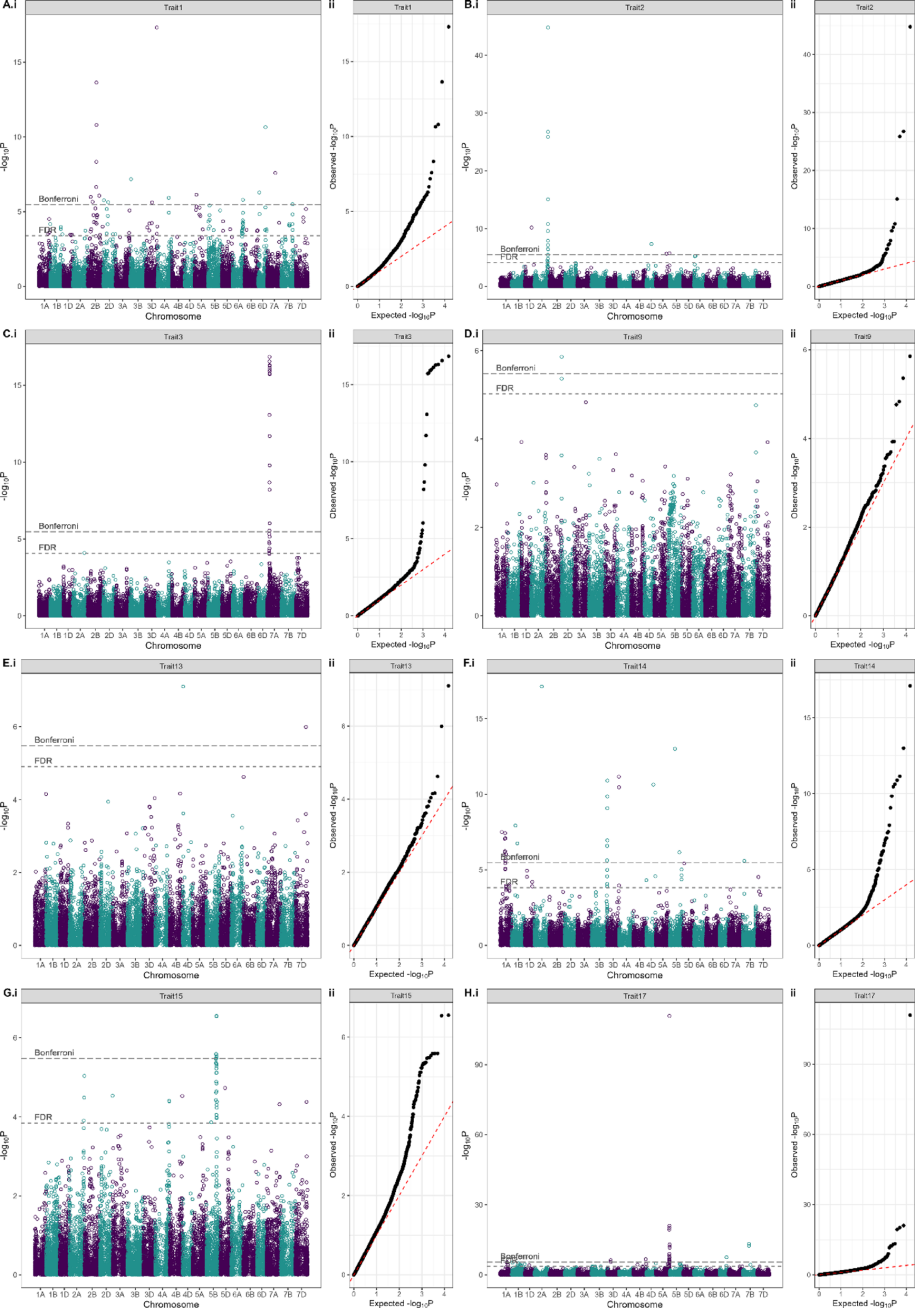

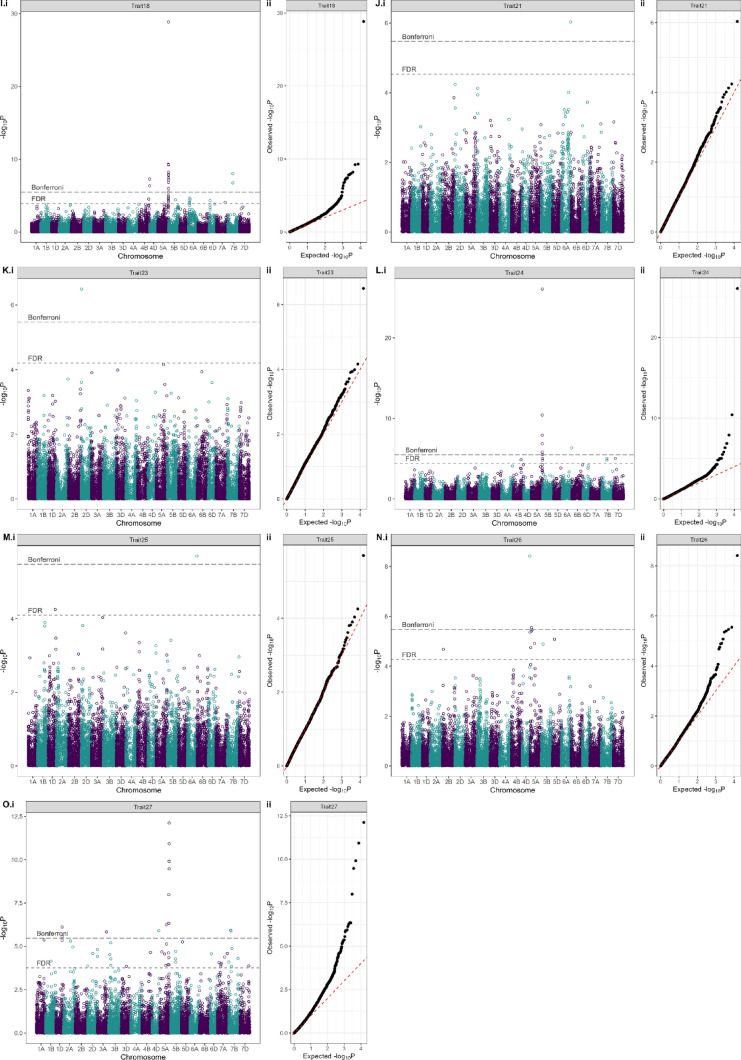
DUS characteristics for which significant (Bonferroni *P* < 0.05) genome-wide association study (GWAS) were identified. Shown are Manhattan plots (left, with the 21 chromosomes of wheat labelled on the x-axis as 1 = chr. 1A, 2 = chr. 1B, 3 = chr. 1D, etc.) and quantile–quantile (QQ) plots (right)

The final data matrix for GWAS consisted of 412 cultivars, 14,921 SNPs, and 24 DUS characteristics. Using a Bonferroni corrected *p* = 0.05 threshold, a total of 152 significant marker–trait associations (MTAs) were identified across 15 of the 24 DUS characters analysed (Fig. 4, Supplementary Table S4, Supplementary Fig. S2). Significant MTAs coalesced into 57 genetic loci (Table 3; Supplementary Table S5). The highest number of loci (14) was identified for Trait 1 (‘seed: colour’), while just one genetic locus was identified for six of the DUS characteristics analysed: Traits 3 (‘coleoptile: anthocyanin colouration’), 9 (‘flag leaf: glaucosity of blade’), 15 (‘ear: density’), 21 (‘apical rachis segment: area of hairiness on convex surface’), 23 (‘lower glume: shoulder shape’), and 25 (‘lower glume: shape of beak’). A total of 11 highly significant genetic loci were identified (defined here as −log_10_P > 12). These included: QTL *Trait1_3D_571* for ‘seed: colour’ (Trait 1) located on chromosome 3D at 571 Mbp, close to the *R1* locus encoded by *TaMYB10-D1* (*TraesCS3D02G468400*, 3D: 571 Mbp) that is known to control grain pigmentation (Himi et al. 2024). QTL *Trait2_2A_713* on chromosome 2A at 712.56 Mb (−log_10_P = 44.79) for ‘seed: colouration with phenol’ (Trait 2), located close to the *Polyphenol oxidase 1* gene (*Ppo-A1, TraesCS2A02G468200,* 2A: 712.19 Mbp) that has previously been associated with browning of wheat-based food products (e.g. He et al. 2007) (Fig. 4b). The highly significant genetic locus *Trait3_7A_121* for ‘coleoptile: anthocyanin colouration’ (Trait 3) on chromosome 7A (−log_10_P = 16.85) located close to the genetic locus *Rc* known to control anthocyanin pigmentation in wheat (Himi & Taketa 2015) (Fig. 4c). Highly significant co-locating QTLs towards the end of the long arm of chromosome 5A for the awn/scur-related DUS characteristics Trait 17 (‘ear: scurs or awns’) (QTL *Trait17_5A_699*, -log_10_P = 110.91) and Trait 18 (‘ear: length of scurs or awns’) (QTL *Trait18_5A_599*, −log_10_P = 28.25), located close the previously cloned *Tipped 1* (*B1*) awning gene (DeWitt et al. 2020; Huang et al. 2020) (Fig. 4h–i). Similarly, QTL *Trait24_5A_699* (− log_10_P = 26.07) for Trait 24 ‘lower glume: length of beak’ was also found close to the *B1 locus* (Fig. 4m). Trait 27 (‘seasonal type’) with highly significant MTAs on chromosome 5A (QTL *Trait27_5A_619*, −log_10_P = 12.11) close to the previously cloned gene *VRN-A1*, for which copy number variation is thought to underlie allelic variation at the associated *Vrn-A1* genetic locus (Díaz et al. 2012) (Fig. 4p). While not significant at −log_10_P > 12, additional GWAS hits for ‘seasonal type’ were located in the regions of previously reported flowering time QTL, including *Trait27_1D_404* (−log_10_P = 6.12) (which co-located with *QFt.niab-1D.03,* and thought to be encoded by *TaELF3-D1*, Fourquet et al. 2024). No significant GWAS hits were identified for nine of the 24 DUS characteristics investigated: Traits 4 (‘plant: growth habit’), 5 (‘plant: frequency of plants with recurved flag leaves’), 7 (‘time of ear emergence’), 8 (‘flag leaf: glaucosity of sheath’), 10 (‘ear: glaucosity’), 11 (‘culm: glaucosity of neck’), 16 (‘ear: length’), 20 (‘ear: shape in profile’) and 22 (‘lower glume: shoulder width’) (Supplementary Tables S1 and S5).

**Table 3:** Summary of genetic loci identified genome-wide association study (GWAS) of DUS characteristics in the ‘INVITE’ wheat association mapping panel

QTL name	Meta-QTL	Trait	Bonferroni *p* = 0.05 sig. threshold	Marker	Chr	Pos. (Mb)^*^	Peri-centromeric	Ref	Alt	− log_10_P	Effect
*Trait14_1A_076*	MQTL_1	Trait14	5.47	*AX-643856718*	1A	76.492	No	0	1	7.50	0.764
*Trait14_1A_253*	MQTL_2	Trait14	5.47	*AX-643798496*	1A	253.363	Yes	0	1	7.42	-0.809
*Trait17_1A_348*	MQTL_2	Trait17	5.47	*AX-643823838*	1A	347.619	Yes	0	1	5.48	-0.422
*Trait14_1B_197*	MQTL_3	Trait14	5.47	*AX-94527213*	1B	197.429	Yes	0	1	7.92	-0.847
*Trait2_1D_348*	MQTL_4	Trait2	5.47	*AX-94574465*	1D	348.034	No	0	1	10.18	-1.065
*Trait27_1D_404*	MQTL_5	Trait27	5.47	*AX-95121546*	1D	403.664	No	0	1	6.12	0.421
***Trait14_2A_386***	MQTL_6	Trait14	5.47	*AX-94402613*	2A	385.699	Yes	0	1	17.11	-0.970
***Trait2_2A_713***	MQTL_7	Trait2	5.47	*AX-94687651*	2A	712.555	No	0	1	44.79	2.031
*Trait1_2B_150*	MQTL_8	Trait1	5.47	*AX-643805636*	2B	150.159	No	0	1	5.99	0.248
***Trait1_2B_450***	MQTL_9	Trait1	5.47	*AX-643843800*	2B	449.599	Yes	0	1	13.64	-0.375
*Trait1_2B_601*	MQTL_10	Trait1	5.47	*AX-95236111*	2B	600.708	No	0	1	6.07	0.127
*Trait9_2D_009*	MQTL_11	Trait9	5.47	*AX-95658025*	2D	9.345	No	0	1	5.86	0.938
*Trait23_2D_031*	MQTL_11	Trait23	5.47	*AX-109760651*	2D	31.118	No	0	1	6.50	1.093
*Trait1_2D_039*	MQTL_11	Trait1	5.47	*AX-643826444*	2D	38.967	No	0	1	5.77	0.216
*Trait1_2D_263*	MQTL_12	Trait1	5.47	*AX-173530267*	2D	262.562	Yes	0	1	5.63	-0.159
*Trait27_3A_608*	MQTL_13	Trait27	5.47	*AX-95014482*	3A	607.967	No	0	1	5.83	-0.449
*Trait1_3B_056*	MQTL_14	Trait1	5.47	*AX-643840750*	3B	55.914	No	0	1	7.18	0.203
*Trait14_3B_830*	MQTL_15	Trait14	5.47	*AX-94434664*	3B	830.227	No	0	1	10.89	-0.568
*Trait17_3D_174*	MQTL_16	Trait17	5.47	*AX-94590365*	3D	173.710	No	0	1	6.29	0.347
*Trait1_3D_324*	MQTL_17	Trait1	5.47	*AX-94756722*	3D	323.719	Yes	0	1	5.62	-0.269
***Trait1_3D_571***	MQTL_18	Trait1	5.47	*AX-94708323*	3D	571.134	No	0	1	17.32	-0.321
*Trait14_3D_602*	MQTL_19	Trait14	5.47	*AX-643840625*	3D	602.265	No	0	1	11.15	0.813
*Trait1_4A_580*	MQTL_20	Trait1	5.47	*AX-643849449*	4A	579.840	No	0	1	5.92	-0.226
*Trait17_4B_649*	MQTL_21	Trait17	5.47	*AX-94404030*	4B	649.475	No	0	1	6.66	0.372
*Trait18_4B_668*	MQTL_21	Trait18	5.47	*AX-95111632*	4B	667.859	No	0	1	7.28	1.146
*Trait13_4D_019*	MQTL_22	Trait13	5.47	*AX-86170701*	4D	18.781	No	0	1	7.10	0.869
*Trait2_4D_271*	MQTL_23	Trait2	5.47	*AX-94417710*	4D	271.272	Yes	0	1	7.34	0.844
*Trait14_4D_373*	MQTL_23	Trait14	5.47	*AX-643815289*	4D	373.454	Yes	0	1	10.62	-0.814
*Trait27_4D_447*	MQTL_24	Trait27	5.47	*AX-94862802*	4D	447.338	No	0	1	5.90	-0.491
*Trait26_4D_503*	MQTL_25	Trait26	5.47	*AX-643804373*	4D	502.728	No	0	1	8.42	-1.051
*Trait26_5A_094*	MQTL_26	Trait26	5.47	*AX-643821605*	5A	94.386	Yes	0	1	5.54	2.035
*Trait27_5A_437*	MQTL_27	Trait27	5.47	*AX-643823902*	5A	437.040	No	0	1	6.25	-0.524
*Trait2_5A_544*	MQTL_28	Trait2	5.47	*AX-94893977*	5A	544.446	No	0	1	5.67	-1.126
*Trait27_5A_587*	MQTL_29	Trait27	5.47	*AX-86178169*	5A	586.605	No	0	1	7.99	0.921
***Trait27_5A_619***	MQTL_30	Trait27	5.47	*AX-643859528*	5A	618.958	No	0	1	12.11	0.739
*Trait2_5A_699*	MQTL_31	Trait2	5.47	*AX-94613491*	5A	698.510	No	0	1	5.75	-1.188
***Trait17_5A_699***	MQTL_31	Trait17	5.47	*AX-94613491*	5A	698.510	No	0	1	110.91	-1.026
***Trait18_5A_599***	MQTL_31	Trait18	5.47	*AX-94613491*	5A	698.510	No	0	1	28.85	-3.167
***Trait24_5A_699***	MQTL_31	Trait24	5.47	*AX-94613491*	5A	698.510	No	0	1	26.07	-2.597
***Trait14_5B_283***	MQTL_32	Trait14	5.47	*AX-95144270*	5B	283.025	Yes	0	1	13.00	0.733
*Trait14_5B_515*	MQTL_33	Trait14	5.47	*AX-643854590*	5B	514.688	No	0	1	6.16	-0.671
*Trait15_5B_515*	MQTL_33	Trait15	5.47	*AX-94758742*	5B	514.853	No	0	1	6.55	-0.680
*Trait1_6A_545*	MQTL_34	Trait1	5.47	*AX-86179563*	6A	545.113	No	0	1	5.79	0.241
*Trait21_6A_588*	MQTL_35	Trait21	5.47	*AX-86185057*	6A	587.778	No	0	1	6.03	0.974
*Trait24_6A_599*	MQTL_35	Trait24	5.47	*AX-94768049*	6A	598.636	No	0	1	6.34	-0.980
*Trait25_6A_599*	MQTL_35	Trait25	5.47	*AX-94768049*	6A	598.636	No	0	1	5.70	-0.869
*Trait1_6D_058*	MQTL_36	Trait1	5.47	*AX-643860229*	6D	57.794	No	0	1	6.28	-0.259
*Trait17_6D_362*	MQTL_37	Trait17	5.47	*AX-95128026*	6D	361.523	No	0	1	7.54	-0.493
*Trait1_6D_382*	MQTL_37	Trait1	5.47	*AX-178060933*	6D	382.245	No	0	1	10.66	-0.331
***Trait3_7A_121***	MQTL_38	Trait3	5.47	*AX-94578662*	7A	121.064	Yes	0	1	16.85	1.929
*Trait1_7A_425*	MQTL_39	Trait1	5.47	*AX-643836974*	7A	424.920	No	0	1	7.59	-0.244
*Trait14_7B_072*	MQTL_40	Trait14	5.47	*AX-643816835*	7B	72.440	No	0	1	5.59	-0.523
*Trait27_7B_128*	MQTL_41	Trait27	5.47	*AX-94712553*	7B	128.070	Yes	0	1	5.93	0.487
***Trait17_7B_315***	MQTL_41	Trait17	5.47	*AX-94881303*	7B	314.915	Yes	0	1	13.26	-0.286
*Trait18_7B_315*	MQTL_41	Trait18	5.47	*AX-94881303*	7B	314.915	Yes	0	1	8.00	-1.175
*Trait1_7B_567*	MQTL_42	Trait1	5.47	*AX-94446867*	7B	586.630	No	0	1	5.51	-0.220
*Trait13_7D_551*	MQTL_43	Trait13	5.47	*AX-643850996*	7D	551.169	No	0	1	5.99	-1.356

Chr. = chromosome. Pos. = position in Mb of genotyped single-nucleotide polymorphism (SNP) in the wheat reference genome assembly of cv. Chinese Spring, RefSeq v1.0 (IWGSC, 2018). Ref. = reference allele, coded as 0, corresponding to the variant present in the wheat reference genome. Alt. = alternative allele, coded as 1

### Conversion of SNP array markers to KASP

**Fig. 5:**
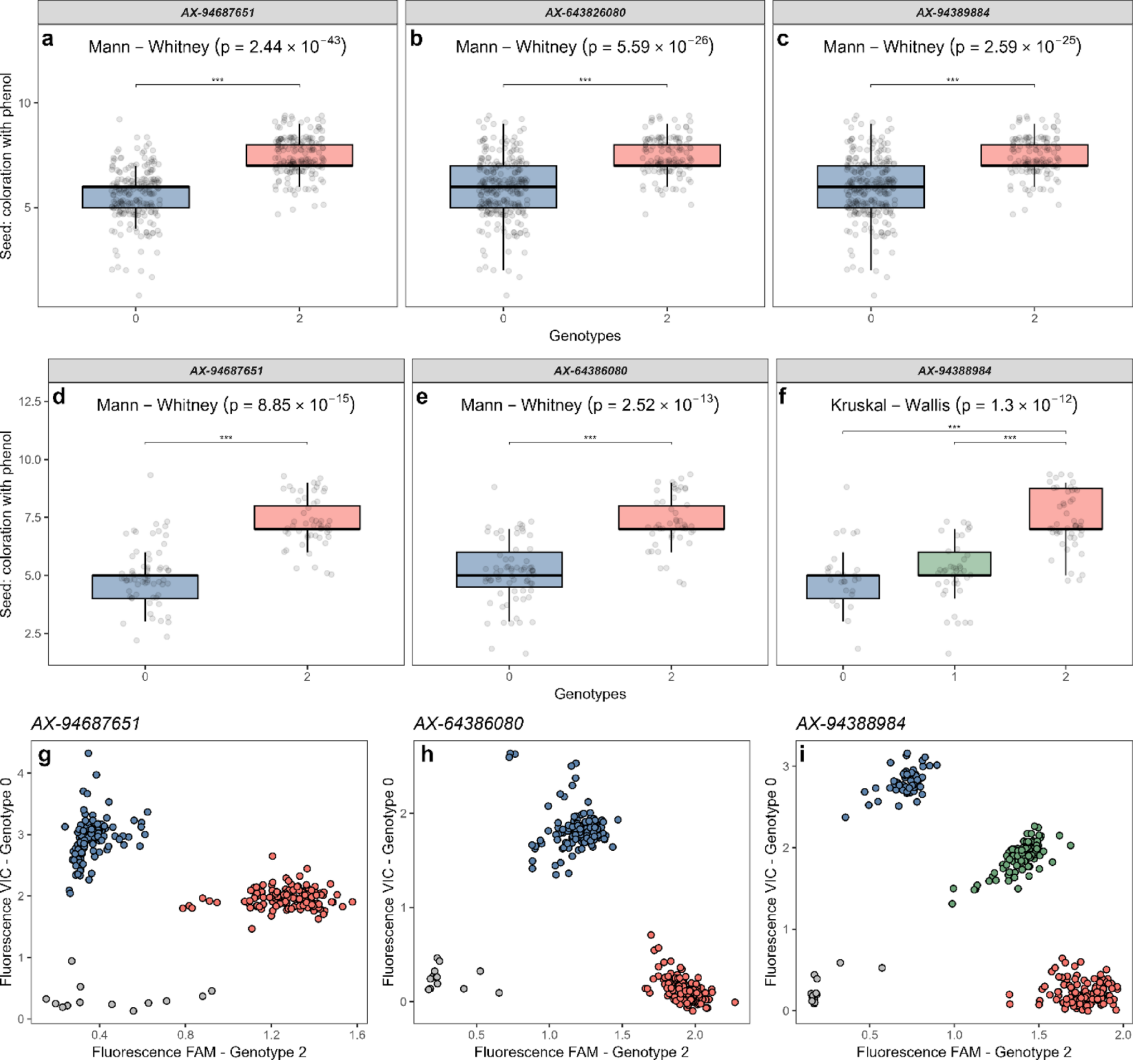
Development and validation of Kompetitive Allele-Specific PCR (KASP) genetic markers for three single-nucleotide polymorphisms (SNPs) identified on the 43k genotyping array as highly associated with the DUS characteristic ‘seed: colouration with phenol’. Boxplots illustrating the association of the trait in the ‘INVITE’ panel with allele calls at 43k array SNPs **a** *AX-643826080*, **b** *AX-94389884* and **c** *AX-94687651*. Similar boxplots illustrating the association of the trait in the independent validation panel of 122 wheat varieties using the developed KASP markers for **d** *AX-643826080*, **e** *AX-94389884* and **f** *AX-94687651*. Significant differences identified via Mann–Whitney test are indicated in the boxplots. Plots showing the fluorescence profiles of the KASP genotyping undertaken in the independent validation panel are shown for each KASP marker, **g** *AX-643826080*, **h** *AX-94389884*, and **i** *AX-94687651*

For a given DUS characteristic, the conversion of significant genetic markers from the 43k SNP array to a genotyping system that allows assessment of single or low numbers of SNP markers at a time would provide greater flexibility for their utilization in downstream applications. Typically, markers for conversion should individually or via haplotype combination with other markers show high association with the target phenotype and allow primer design that allow target-specific amplification. To illustrate this, we converted the most significant markers at genetic locus *Trait2_2A_713* for the DUS characteristic ‘seed: colouration with phenol’ (Trait 2) to the KASP genotyping platform. First, we used the 43k SNP array genotypic data to construct haploblocks across the target genetic locus. Analysis of haplotypes within the target haploblock a haplotype-based approach did not improve prediction based on SNP *AX-94687651* alone (data not shown). Nevertheless, we designed KASP assays for *AX-94687651* as well as the next two most significant markers (*AX-643826080* and *AX-94389884*) and used these to genotype an independent validation panel of 122 wheat cultivars (Supplementary Table S6) for which existing DUS data for Trait 2 were available. For all three KASP markers, the resulting genotypic data confirmed that a T SNP (coded as genotype = 2, assayed using the red FAM fluorophore) was predictive of darker seed discolouration on treatment with phenol in the validation panel, while a C SNP (coded as genotype = 0, assayed using the red FAM fluorophore) was predictive of lighter seed discolouration (Mann–Whitney test, *p* ≤ 2.08 × 10^–28^) (Fig. 5).

## Discussion

### SNP variation within the ‘INVITE’ wheat association mapping panel

To our knowledge, this is the first investigation to publish results of the TaNG 43k SNP array beyond that described in the publication of the array itself (Burridge et al. 2024). The TaNG array was specifically designed to address issues with previous wheat SNP arrays, including uneven marker distribution due to linkage disequilibrium, and the use of limited genetic diversity for the prior identification of SNPs to be assayed. The ‘INVITE’ association mapping panel assembled here (*n* = 412) sampled genetic diversity from elite wheat cultivars released in five European countries, ranging from north-western regions (UK), through central (Germany, Austria) and eastern (Hungary) Europe, to southern Europe (Italy). We found 45% of the SNPs assayed by the TaNG v1.1 array to be polymorphic (19,602 of 43,472) in our association mapping panel. This compares favourably to the 47% (20,507 of 43,472) of TaNG array SNPs identified as polymorphic in the panel of 398 wheat genotypes assessed by Burridge et al. (2024), which included cultivars from 19 countries as well as 22 landraces from around the world. Thus, based on the variation assayed by the TaNG 43k array, the ‘INVITE’ association mapping panel captured a large proportion of the SNP variation found in the wider wheat genepool assessed by Burridge et al. (2024). Previous wheat SNP genotyping arrays suffered from a relative lack of genetic markers on the wheat D sub-genome, due to (i) ascertainment bias (Burridge et al. 2024), and (ii) the genetic bottleneck caused by the relatively recent speciation of bread wheat that occurred around the Caspian Sea via natural hybridization between AB and D genome wheat species (e.g. Gaurav et al. 2022). This has resulted in potential issues for the identification of genetic loci in wheat via GWAS (e.g. Maccaferri et al. 2015). The TaNG v1.1 array was designed to include roughly equal proportions of SNP numbers across the wheat sub-genomes, with 31% of the designed markers located on the D sub-genome (corresponding to 29% of the SNPs successfully genotyped here). The more balanced nature of the TaNG SNPs between the sub-genomes (and within chromosomes) is reflected in part via roughly equal rates of linkage disequilibrium decay observed between sub-genomes in the ‘INVITE’ panel (Fig. 1). This contrasts with the lower levels of decay reported on the D sub-genome in comparison to the A and B sub-genomes for wheat cultivars genotyped with previous SNP arrays (e.g. Gardner et al. 2025).

### DUS phenotypic variation within the ‘INVITE’ wheat association mapping panel

DUS assessment is at the heart of obtaining Plant Breeders’ Rights, with DUS accreditation also being a pre-requisite for the commercial sale of wheat varieties in some countries. Good levels of variation were observed for the majority of wheat DUS characteristics analysed in the ‘INVITE’ panel. However, some characteristics showed extremely limited phenotypic variation, while others showed low heritability. Both findings have implications for DUS processes. If DUS characteristics are to be fit for their intended purpose, variation must be present in the current breeding genepool and the results must be reasonably repeatable between seasons and trial locations. We found significant (*p* < 0.05) correlation between DUS characteristics to be frequent. Some correlations were as might be expected a priori, for example: (i) the positive correlation between the presence of awns and the length of the glume beak from which the awn extends, (ii) the positive correlations between the levels of glaucosity on different plant tissues, and (iii) the negative correlation between ear length and spikelet density. Of note was the finding that winter seasonal growth habit was associated with a more prostrate growth habit, which may help define ideal wheat ideotypes. While not directly assessed here, correlations between DUS characteristics and agronomic traits under strong breeder selection may result in direct or indirect selection for specific combinations in DUS trait space. For wheat, the primary breeding targets are for high yielding cultivars with good end-use properties (for example, bread making, biscuit making, or animal feed) and resistance to disease (Reif et al. 2011). Distinctions in wheat end-use may have affected the observed phenotypic variation for ‘seed: colour’ as red grain is associated with bread-making end-use quality while white grain is associated with biscuit making (Subedi et al. 2023). Although phenotypic data for ‘seed: colour’ were available for less than 100 cultivars, it was notable for being one of the three wheat DUS characteristics showing very low trait variation. Almost all cultivars were scored as having a grain colour of ‘reddish’, along with 4% scored as ‘white’, with no instances of scores reflecting higher levels of grain pigmentation—potentially reflecting bias due to intended end-use in our European wheat panel. Similarly, the almost complete lack of variation in observed pigmentation for ‘ear: colour’, whereby 99% of cultivars were recorded as ‘white’, may also reflect the association between the plant’s ability to produce pigmentation in the ear/grains and end-use quality. Interestingly, correlation between DUS characteristics and yield in the related cereal crop barley has reduced DUS characteristic combinatorial space over time due to breeder selection for increased yield (Yang et al. 2021). The barley DUS traits showing such trends (‘plant: length’ and ‘ear: attitude’) are likely have a relatively direct effect on yield components, thus helping to define the ideal barley ideotype (Yang et al. 2021). The DUS characteristic ‘lower glume: hairiness on external surface’ was the only other wheat DUS characteristic with limited observed phenotypic variation in the ‘INVITE’ panel, with all but one of the cultivars for which data were available recorded as glume hair ‘absent’. Major effect genetic loci controlling glume hairiness have been identified, including *Hairy glume* (*Hg*) on the short arm of chromosome 1A at around 1–2 Mb (Luo et al. 2022) and *Hg2* on the long arm of chromosome 2B at around 755 Mb (Wu et al. 2021). While *Hg2* is located by GWAS close to a previously identified QTL for yield in a panel of UK wheat varieties (*YLD_2B.4,* chromosome 2B at 766 Mb; White et al. 2022), the origin of *Hg2* from synthetic hexaploid wheat makes it less likely to have been present in the European wheat genepool. While glume hairiness does not have clear direct links to major wheat breeding traits, the presence of glume hairs is anecdotally associated with increased ear mildew infection (Phil Howell, pers. comm.), could be associated with post-harvest handling challenges, and have more recently been identified as a potential health risk (Lian et al. 2019). Given the dominant major semi-dominant effect nature of the known *Hg* and *Hg2* genetic loci, intentional breeder selection against glume hairiness would likely have been easy to achieve via visual selection—and could even have been an instinctive breeder choice to make the wheat ears appear more visually appealing.

### DUS marker–trait associations

GWAS identified significant hits for two thirds of the wheat DUS characteristics investigated, providing baseline genetic data for future investigations. The two most significant GWAS hits were associated with the known genetic loci *B1* (which controls awn presence/absence. Huang et al. 2020) and *Ppo-A1* (which controls wheat product discolouration. He et al. 2007). For DUS characteristic ‘ear: scurs or awns’, the SNP *AX-94613491* located at 698.510 Mb on chromosome 5A correctly discriminated between awned versus non-awned/scurs in all but five cultivars and was located 190 kb from the *B1* gene (*TraesCS5A02G542800*, a C2H2 transcription factor that represses the awn phenotype. Chr. 5A at 698.529 Mb). As *B1* copy number variation (CNV) is thought to control awn phenotype (DeWitt et al. 2020; Li et al. 2023), linked SNPs such as that identified here could be preferable for molecular assessment than a CNV assay—which are typically more costly to implement. GWAS for ‘seed: coloration with phenol’ identified marker–trait associations at genetic locus *Trait2_2A_713* within 0.37 Mb of the *Ppo-A1* gene. Although the SNP identified here was highly significant (*AX-94687651*, −log_10_P = 44.79) and was shown to be convertible to the KASP genotyping platform, it was not able to discriminate diagnostically between the two extremes of the trait expression (i.e. between the absence of discolouration versus very dark discolouration) in either the ‘INVITE’ panel or the independent panel of cultivars used here to validate the KASP marker. This could be due to segregation at the four additional genetic loci identified controlling the trait (*Trait2_1D_348*, *Trait2_4D_271*, *Trait2_5A_544*, *Trait2_5A_699*) or due to additional structural variation at the target locus not immediately apparent from the use of the genotyping array data for single marker or haplotype analysis. Notably, while the KASP markers developed here result in clear allele clusters, there is evidence of co-amplification of additional copies of the allele that results in darker seed colouration on phenol treatment: for the most significant marker identified via GWAS (*AX-94687651*), this is evident in the KASP data from the allele associated with darker seed colouration (genotype call 2, x-axis, red) being plotted at relatively high fluorescence intensity on the y-axis (which measures blue intensity associated with genotype call 0) (Fig. 5i). For KASP marker *AX-643826080*, this phenomenon is inverted, with greater baseline fluorescence intensity on the x-axis (Fig. 5g). Finally, for KASP marker *AX-94389884* (Fig. 5h) the situation is less clear: there is evidence of co-amplification for some cultivars (green cluster, classically termed heterozygous) but not others, with association with phenotype indicating no statistically significant difference between the ‘heterozygous’ class and the homozygous allele 0 class. As the cultivars assayed are inbred lines, and the KASP assay was designed to be homoeologue specific, taken together with the results from the other two KASP markers and 43 l array-based haplotype analysis, these results raise questions as to whether copy number variation may play a role at this locus. Interestingly, numerous *Ppo-A1* alleles have been reported (e.g. He et al. 2009), which would impact the ability of any single genetic marker to predict phenotype. Co-dominant genetic markers have previously been developed that are able to distinguish between the wild-type *Ppo-A1a* allele and the null *Ppo-A1b* and *Ppo-A1i* alleles which confer reduced darkening of wheat flour and noodle products (Heesacker et al. n.d.; Nakamaru et al. 2023). Future work including genotyping the ‘INVITE’ panel with such markers, as well as more detailed haplotype and genomic analysis around the *Ppo-A1* locus, could help refine the marker–trait associations at the *Trait2_2A_713* locus and the nature of the underlying genes and variants controlling this trait. Finally, while the homologous *Ppo-D1* gene and paralogous *Ppo-A2, Ppo-B2* and *Ppo-D2* genes have been reported as modulating wheat product discolouration (e.g. Beecher et al. 2012), the absence of GWAS hits at these loci indicates they do not contribute to the seed discolouration trait in the European wheat germplasm investigated here.

No significant (Bonferroni corrected *p* > 0.05) GWAS hits were identified for nine of the DUS characteristics investigated. For these, lack of marker–trait associations could be due to several reasons, including insufficient association mapping panel size, complex underpinning genetic architecture, and low heritability (Cockram & Mackay 2018). The finding that GWAS hits were identified using the less stringent FDR *q* = 0.05 significance threshold in four of these eight traits (Supplementary Fig. S2) indicated that increasing the size of the association mapping panel to improve QTL detection power would likely help address the issue. As mean heritability for the remaining five characteristics (‘time of ear emergence’, ‘flag leaf: glaucosity of sheath’, ‘ear: glaucosity’, ‘ear: shape in profile’, and ‘lower glume: shoulder width’) was low (*H*^*2*^ = 0.30) compared to that across all DUS traits (*H*^*2*^ = 0.44), a large amount of their phenotypic variation is not genetically controlled, indicating that their further genetic analysis could be problematic. Similar issues have been reported for DUS characteristics in barley (Yang et al. 2021). The principal routes to enhancing the probability of detecting significant marker–trait associations in future research would be to increase statistical power and trait heritability. This could be done via the inclusion of more cultivars in the association mapping panel, greater replication of cultivars within DUS assessment trials, or by improving the accuracy of DUS characteristic phenotypic measurements. However, if the genetic component of a particular DUS characteristic is inherently low, while such measures may help detection of at least some of the underpinning genetic loci, ultimately the effect of the environment may play a predominant role on phenotypic expression.

### Potential implications

The molecular and genetic datasets generated here are of potential use for a variety of downstream applications. For those DUS characteristics with high heritability and which were found to be controlled by a small number of highly significant genetic loci, the conversion of SNPs from the TaNG genotyping array to low- to medium-throughput platforms such as Kompetitive Allele-Specific PCR (KASP), or the validation of previously published molecular assays for genes such as *Ppo-A1* found to potentially underlie DUS GWAS hits identified here, could allow future development of like-for-like replacement of visual assessment with a molecular marker assay. For DUS characteristics with complex genetic architecture and/or low heritability, like-for-like replacement with genetic markers is not feasible, and the datasets assembled here could be used to explore genomic prediction-based approaches (e.g. Crossa et al. 2017; Montesinos López et al. 2022) to predict DUS phenotype that incorporate suitable thresholds for the calling of Distinctness. Within DUS testing, potential new ‘candidate’ cultivars must be phenotypically assessed for DUS characteristics alongside ‘common knowledge’ varieties that form the ‘Reference Collection’ across at least two field seasons. The genetic markers and genetic knowledge generated here could be used to streamline the DUS testing process, for example by improving the selection of Reference Collection varieties for side-by-side DUS comparison. Additionally, DUS Test Centres validate the identity of seed samples that will be included in the variety collection or in some cases (for example in Variety Listing in the UK) to confirm that seed submitted for Value for Cultivation and Use (VCU) testing is the same variety as that used for DUS testing. These are primarily carried out via protein electrophoresis or visual comparison of side-by-side field plots. The dataset generated here would allow future design of ‘minimal molecular marker sets’ for cultivar identification in such contexts.

## Supplementary Information

Below is the link to the electronic supplementary material.

Supplementary file1 (XLSX 18871 KB)

Supplementary file2 (DOCX 2045 KB)

## Data Availability

The genotypic datasets analysed during the current study are available in the Supplementary Files, using anonymized codes in place of variety names. The DUS phenotypic datasets are available from the corresponding author on request and with confirmation from the relevant breeding companies of permission for further distribution.
