# Supplementary material for: Genome-wide association study (GWAS) identifies genetic loci controlling Distinctness, Uniformity, and Stability (DUS) traits in wheat
Source: Theor Appl Genet. 2026 Jan 28;139(2):49. doi: 10.1007/s00122-025-05130-4 (PMC12852309; doi:10.1007/s00122-025-05130-4)
Supplement: Supplementary file 2 — Supplementary file2 (DOCX 2045 KB) [file 122_2025_5130_MOESM2_ESM.docx]

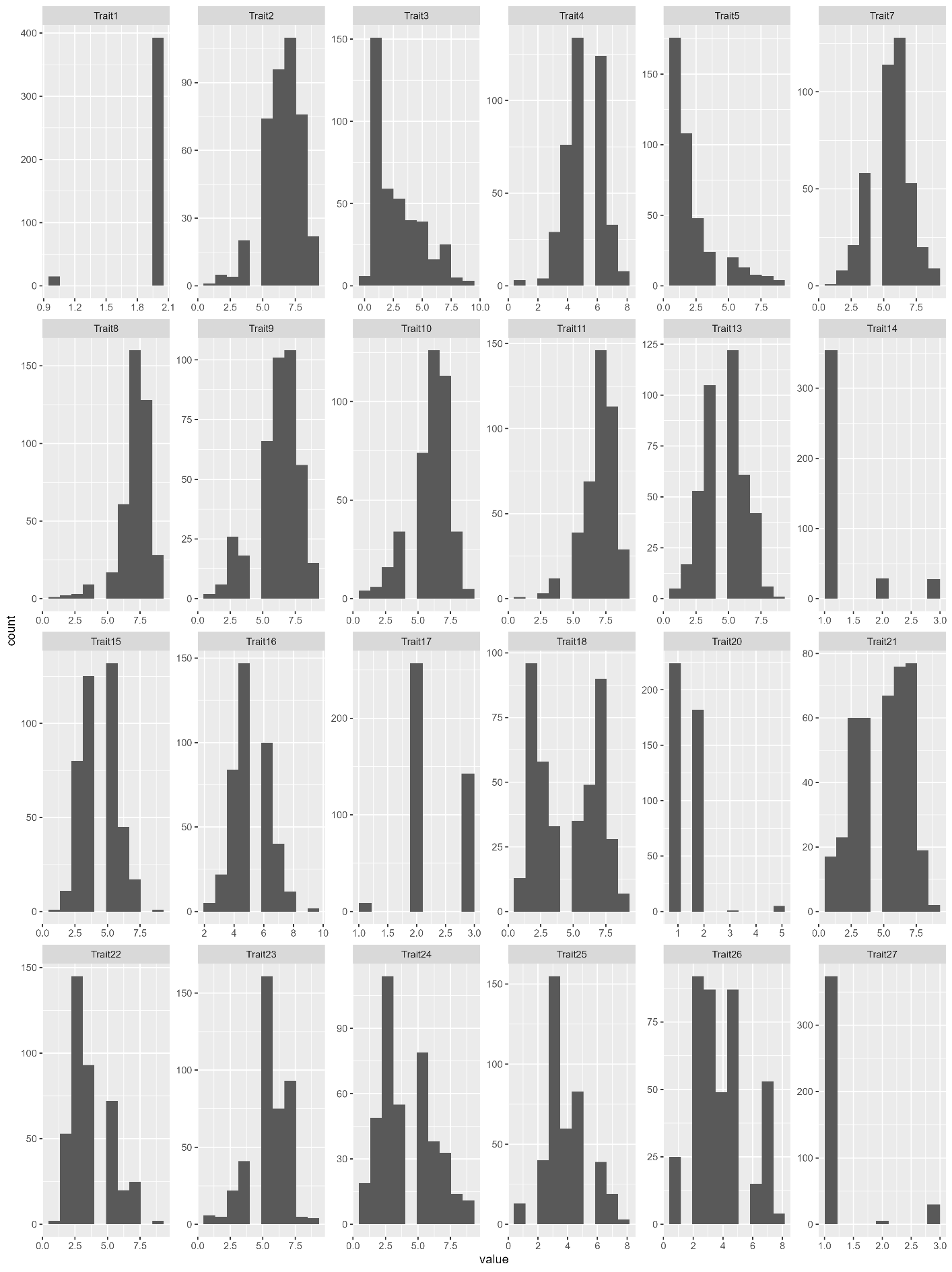
 **Supplemental Figure S1.** Histograms of phenotypic scores for the 28 DUS characteristics for which data was sourced from the INVITE wheat panel.


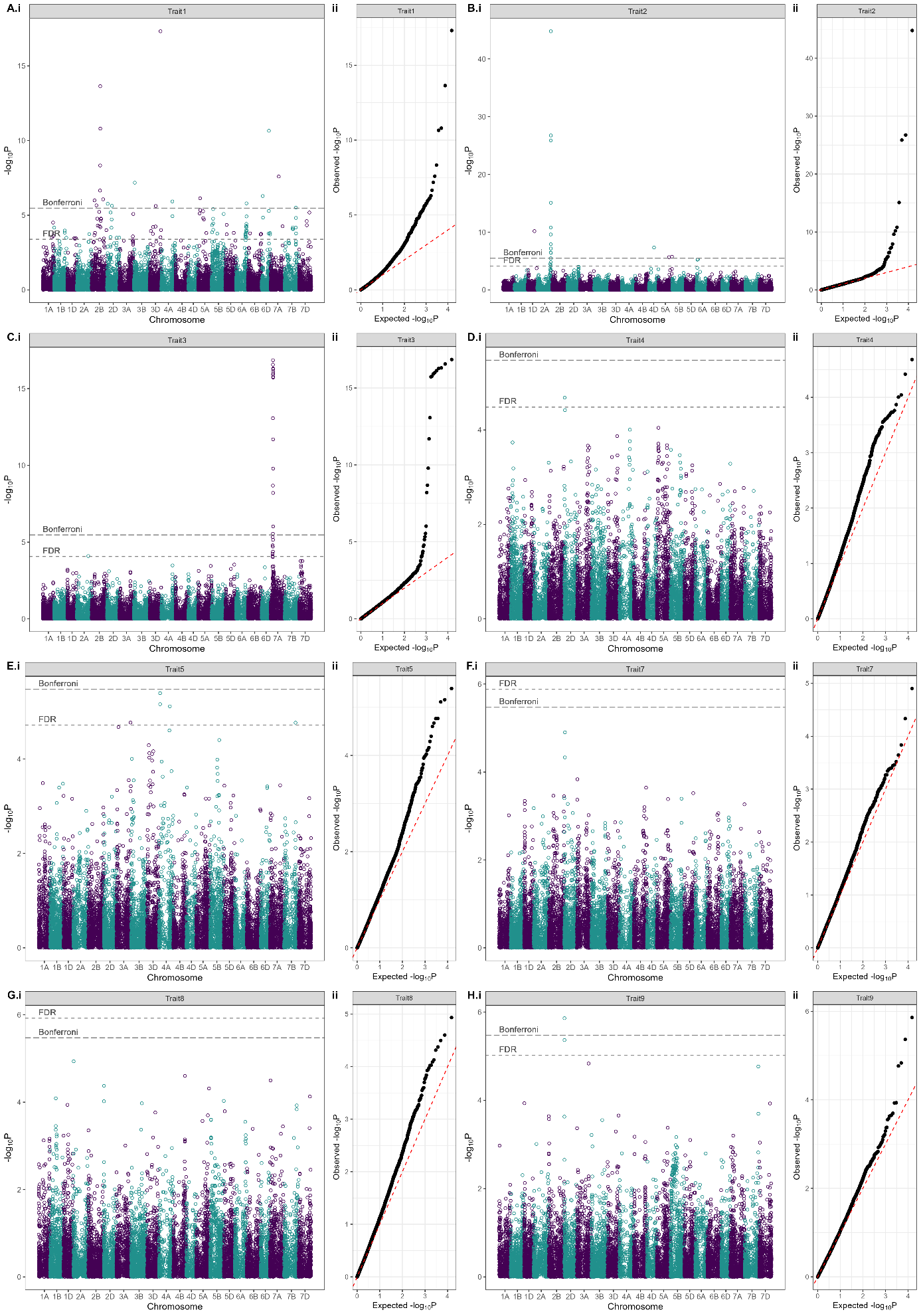


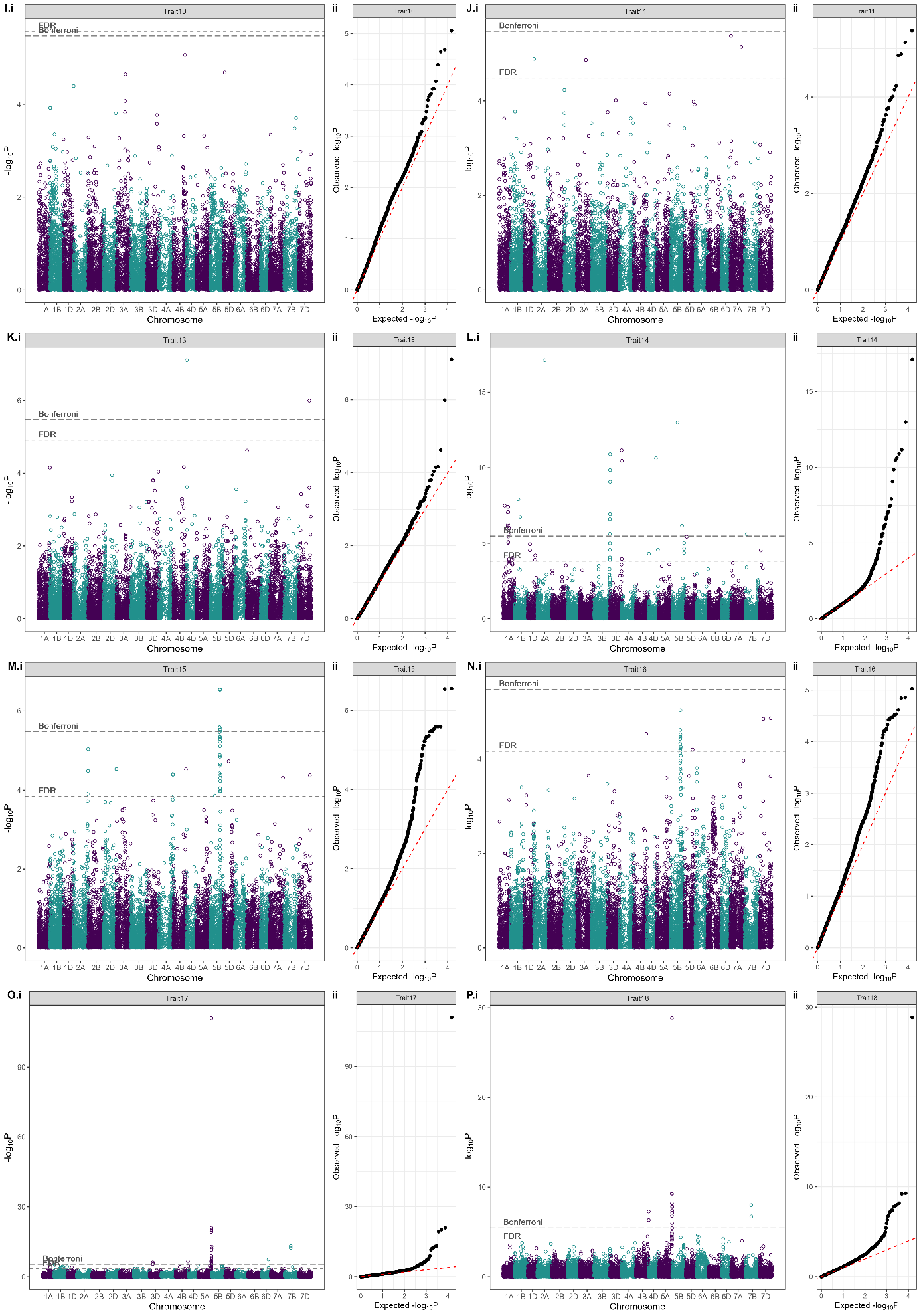


**
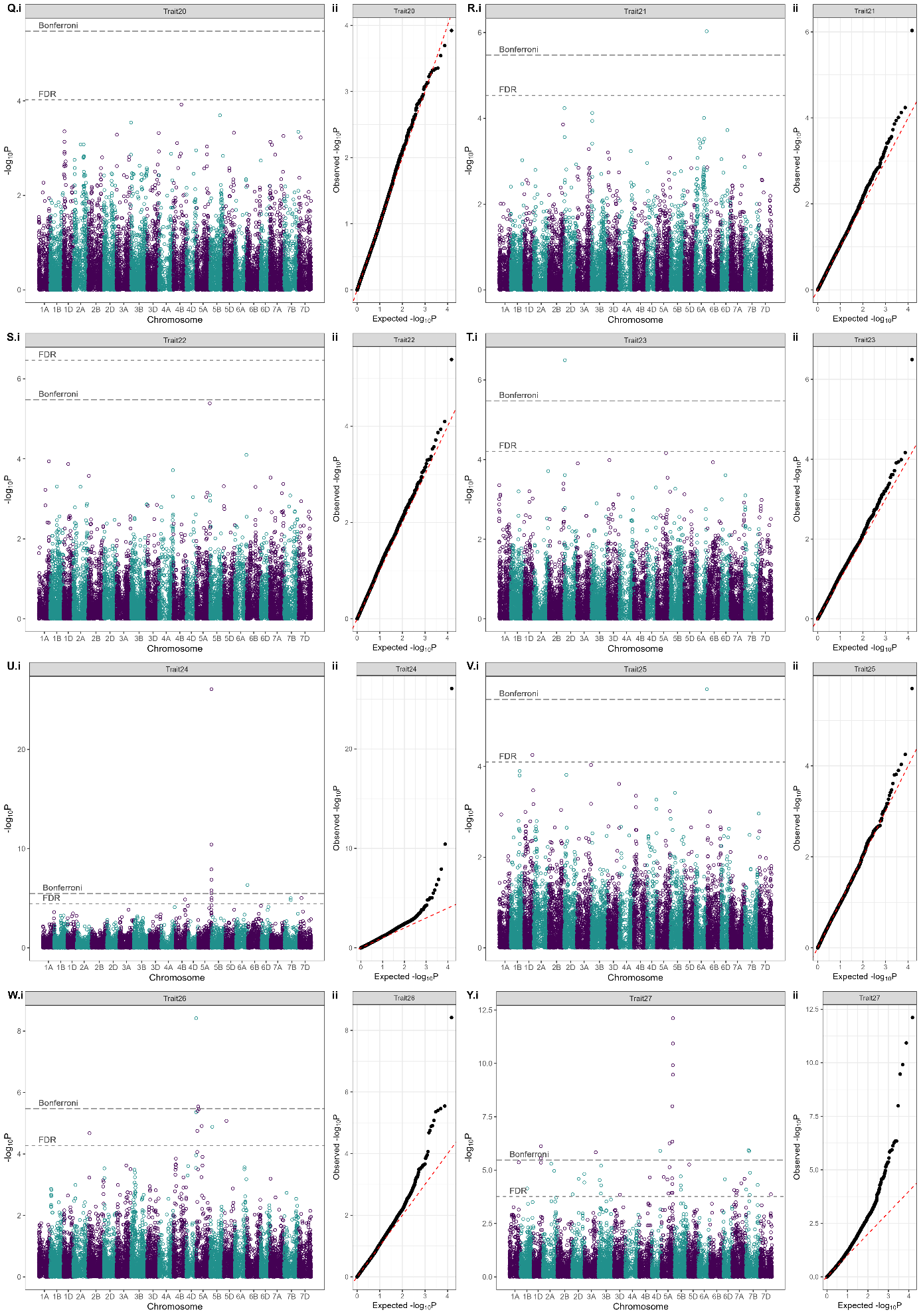
Supplemental Figure S2.** Genome wide association study (GWAS) results for all 24 wheat DUS characteristics analysed. Manhattan plots (left) which include the Bonferroni P < 0.05 and false discovery rate (FDR) *q* = 0.05 significance thresholds. Quantile-quantile (QQ) plots (right) are also shown.
